# Case Report: Endoscopic lumbar interbody fusion using percutaneous unilateral biportal endoscopy and 3D printing for an andersson lesion in ankylosing spondylitis

**DOI:** 10.3389/fsurg.2025.1428072

**Published:** 2025-01-20

**Authors:** Xiaofeng Yuan, Rui Tao, Mengfei Zhu, Jiajun Zhu

**Affiliations:** Department of Spine Surgery, The Third Affiliated Hospital of Soochow University, Changzhou, China

**Keywords:** andersson lesion (AL), ankylosing spondylitis (AS), unilateral biportal endoscopy (UBE), lumbar interbody fusion (LIF), three-dimensional (3D)

## Abstract

**Background:**

An Andersson lesion (AL) is a late-stage lesion of ankylosing spondylitis (AS) that can be misdiagnosed. If the patient has unbearable pain or symptoms indicative of neurological damage, then posterior fusion can be considered. Compared with open surgical procedures, combining Unilateral biportal endoscopy (UBE) and 3D-printing technologies for endoscopic lumbar interbody fusion (LIF) can offer the advantages of minimal trauma and the same effect. In this study, we first used UBE with endoscopic LIF for an AL between T12 and L1 in a 43-year-old male patient with good clinical outcomes.

**Methods:**

A 43-year-old man was admitted to our hospital due to recurrent back pain for 8 years. Based on imaging (computed tomography and radiography) findings, medical history, and clinical examination, we carried out an HLA-B27 blood test to confirm the diagnosis of AS with AL. Finally, we undertook fully endoscopic LIF with UBE based on a 3D-printing model. This patient's pre- and postoperative radiological and clinical results were presented.

**Results:**

Accurate preoperative planning based on a 3D-printing model is strongly recommended for patients with an AL who have ambiguous anatomic landmarks. Applying endoscopic techniques and 3D-printing technologies to the surgical treatment of AL is completely feasible and has an edge in terms of tissue damage.

**Conclusion:**

Endoscopic LIF with UBE based on a 3D-printing model showed a favorable clinical and radiological result and appears to be a safe and effective technique for an AL.

## Introduction

An Andersson lesion (AL) is a rare complication of ankylosing spondylitis (AS). It was first described by Andersson in 1,937 (1). Clinical symptoms manifest as persistent pain in the lower back, with obvious tenderness at the spinous process upon physical examination. Few patients with an AL will have symptoms of compression of dura mater or nerves caused by fracture displacement. Often, an AL is misdiagnosed as purulent spondylitis or spinal tuberculosis, and the treatment for these diseases varies considerably. Therefore, an accurate diagnosis is of paramount importance. Many patients may experience persistent dull pain and can be treated conservatively. For those with severe pain or neurological symptoms, surgical treatment is recommended to rebuild spinal stability and relieve nerve compression. The surgical techniques employed are varied and encompass open fixation, open fixation with osteotomy, open fixation with lumbar interbody fusion (LIF), and minimally invasive surgery (MIS) fixation with and without LIF. Here, we report an atypical case of a 43-year-old man diagnosed with a neglected AL. We completed LIF with unilateral biportal endoscopy (UBE) and fixation using percutaneous pedicle screws (PPSs).

## Case presentation

**Figure 1:**
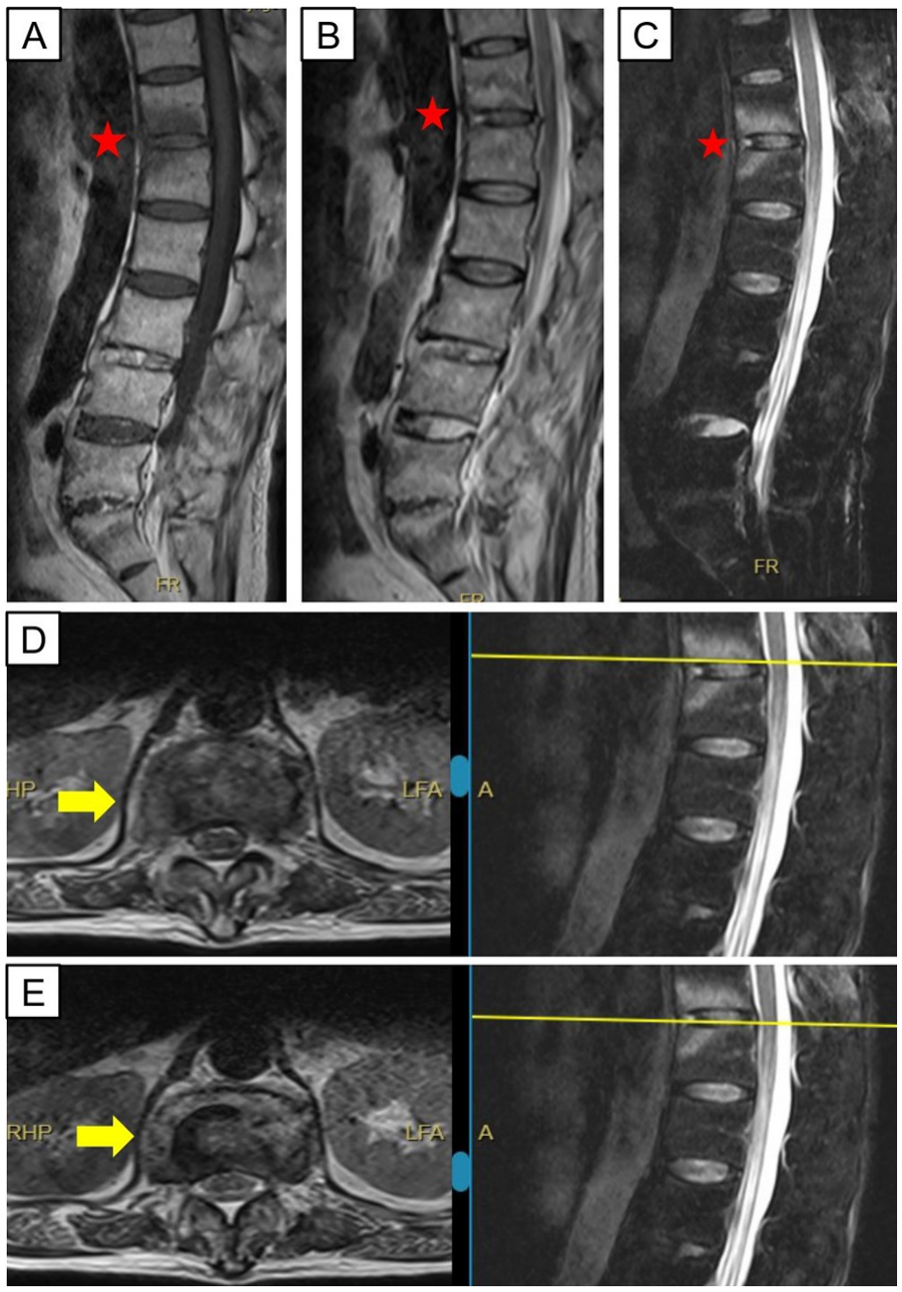
Magnetic resonance images of the patient before admission. The red pentagrams indicate the lesion mimicking ModicⅠchanges, which are hypointense on T1-weighted sequences **(A)**, hyperintense on T2-weighted sequences **(B)** and STIR sequences **(C)** Yellow arrows show inhomogeneous signal in the disc space at T12/L1 in keeping with discitis **(D,E)**.

A 43-year-old man complaining of lower back pain that had persisted for 8 years was admitted to our hospital. The progressively increasing pain and unresponsiveness to analgesics or physiotherapy based on traditional Chinese medicine encouraged him to seek additional treatment in our hospital. The patient appeared to be physically healthy. He was not using medication, had not undergone surgery, and did not have inherited diseases in his family. He had not experienced fever, weight loss, coughing, or night sweats. At the initial visit, the patient only had magnetic resonance imaging (MRI) data from a different hospital, which showed endplate erosion and damage to intervertebral disks (Figure 1). Physical examination showed no other signs of nerve damage except for obvious tenderness at the spinous process of the lower back with limited activity upon flexion and extension. Based on symptoms and imaging data, we preliminarily suspected spinal infection, and he was admitted to our hospital.

**Figure 2:**
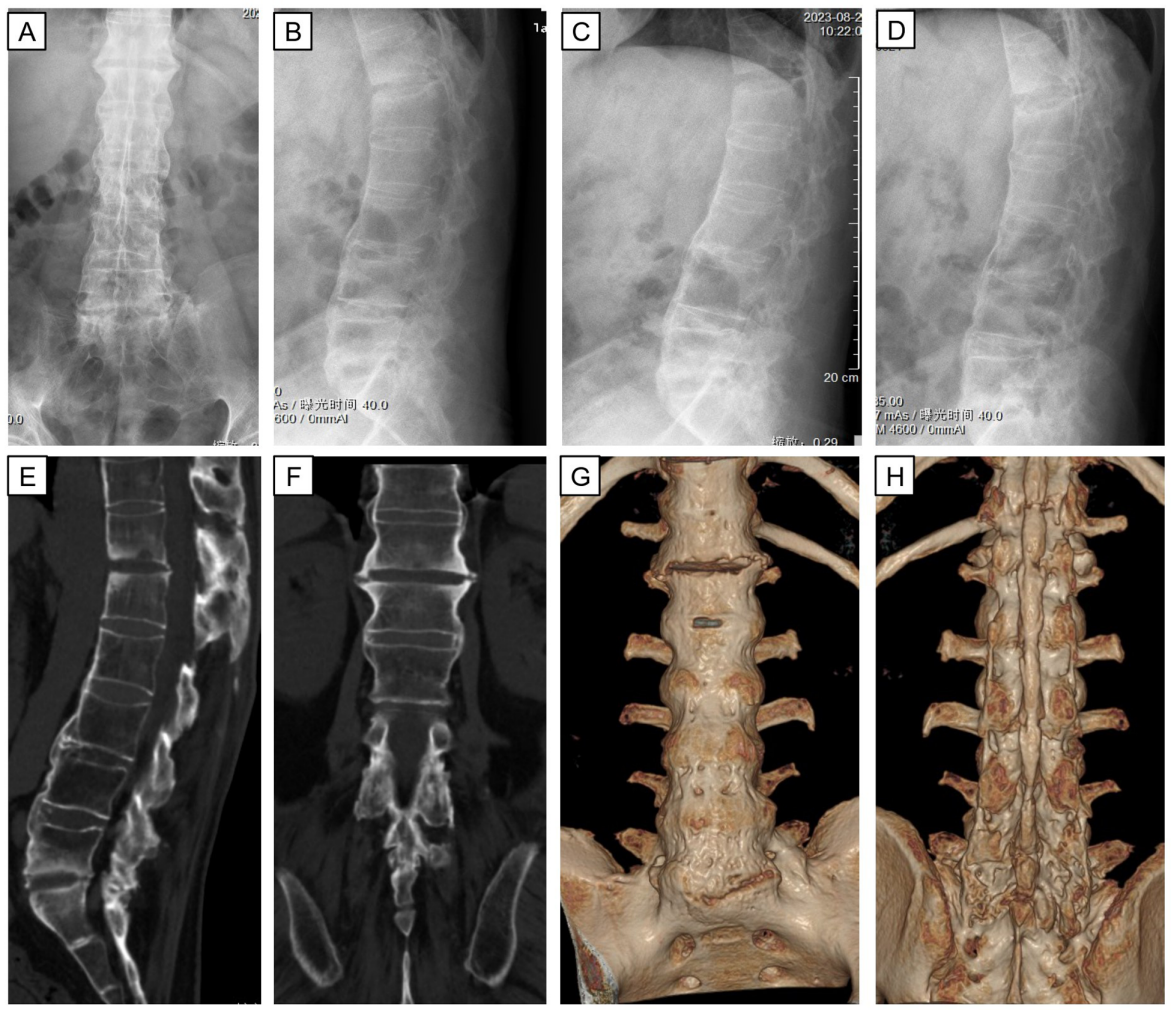
X-ray and CT three-dimensional reconstruction of thoraco-lumbar vertebrae after admission. Anterior and lateral radiographs **(A,B)** show abnormal paravertebral and anterior longitudinal ligament ossification. Flexion and extension lateral radiographs **(C,D)** represent a loss of lumbar mobility. Sagittal, coronal, and reconstructed coronal CT images **(E–H)** demonstrate instability of the lumbar spine result from no bone bridging between T12 and L1.

Blood samples for hematologic indices and infection-related indices were collected as part of the routine examination after admission (Table 1). We found no abnormalities except for a slightly higher erythrocyte sedimentation rate (ESR). Therefore, we could exclude bacterial or viral infections. Radiography showed separation of T12-L1 vertebrae and sclerotic endplates in the spine. Computed tomography (CT) showed corresponding widening of the intervertebral space, the “vacuum sign” in the joint space, destruction of endplate bone, and peripheral-bone sclerosis. Based on imaging (CT and radiography) findings (Figure 2), medical history, and clinical examination, we performed an HLA-B27 blood test to confirm the diagnosis of AS with an AL.

**Table 1:** Laboratory test indicators during the hospitalization of this patient.

Variable	Value	Measure	Normal range
WBC	8.52	×10^9/L	3.5–9.5
Neutrophils	65	%	40–75
Eosinophils	2.8	%	0.4–8
Basophils	0.5	%	0–1
lymphocytes	25.6	%	20–50
Monocytes	6.1	%	3–10
CRP	8	mg/L	<10
ESR	39↑	mm/h	<21
PCT	0.196	ng/ml	0.021–0.5
Tubercle bacillus antibody	Negative	-	Negative
T-spot	Negative	-	Negative
Sputum smear for acid-fast bacilli (AFB)	Negative	-	Negative
G test	Negative	-	Negative

**Figure 3:**
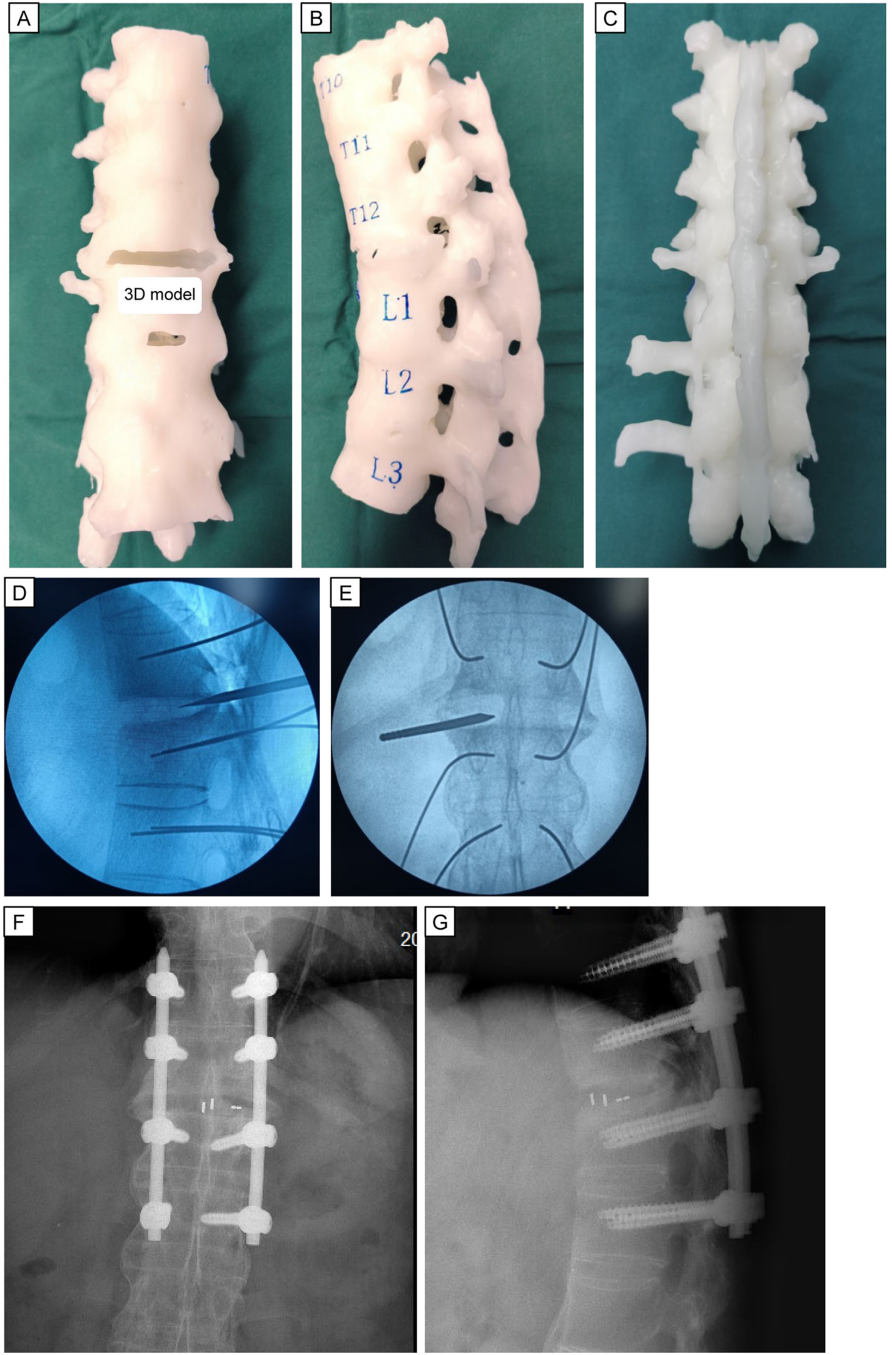
Preoperative 3D model printing and intraoperative fluoroscopic images. Anterior **(A)**, lateral **(B)**, and posterior **(C)** view of the 3D printed model. Intraoperative anterior and lateral radiographs (**D,E)** demonstrate the proper position for the diseased lesion using location-needle. Anterior and lateral radiographs **(F,G)** of excellent fusion cage and percutaneous pedicle screws placement is seen.

After communication between the treating physicians and patient, the latter refused the original plan of oral intake of non-steroidal anti-inflammatory drugs (*N*SAIDs) and immunosuppressive drugs. Sticking to medication and physiotherapy did not get rid of his back pain. On the contrary, the patient is still unable to carry out long-term physical activities every day. So he requested surgical treatment. Considering the minimally invasive requirements of the patient, we adopted the fully endoscopic LIF procedure with UBE. Under general anesthesia, the patient was placed prone on a radiolucent operating table. Precise preoperative planning can be aided by 3D printing technology. Considering the difficulty in inserting screws caused by many posterior osteophytes, we designed the insertion positions, angles, lengths of eight pedicle screws, and curvature and length of the titanium rods based on a 3D-printing model in advance. Simultaneously, stiffness and deformity in the back of the patient hampered threading of the long titanium rods. We could also determine the curvature and length of the titanium rods based on a 3D-printing model. This preoperative planning based on a 3D-printing model ensured the safety of instrumentation placement and decompression process, and it also shortened the duration of the surgical procedure significantly. Eight pedicle guidewires were inserted sequentially into the T11-L2 pedicle using G-arm fluoroscopy. Subsequently, decompression and interbody fusion were completed under endoscopic monitoring. The position of the intervertebral fusion cage was identified by G-arm fluoroscopy. Then, eight PPSs were inserted (Figure 3). Finally, we used two pre-bent titanium rods to connect the pedicle screws. After confirming the position of the internal fixation position again with G-arm fluoroscopy, we placed a drainage tube and sutured the wound.

**Figure 4:**
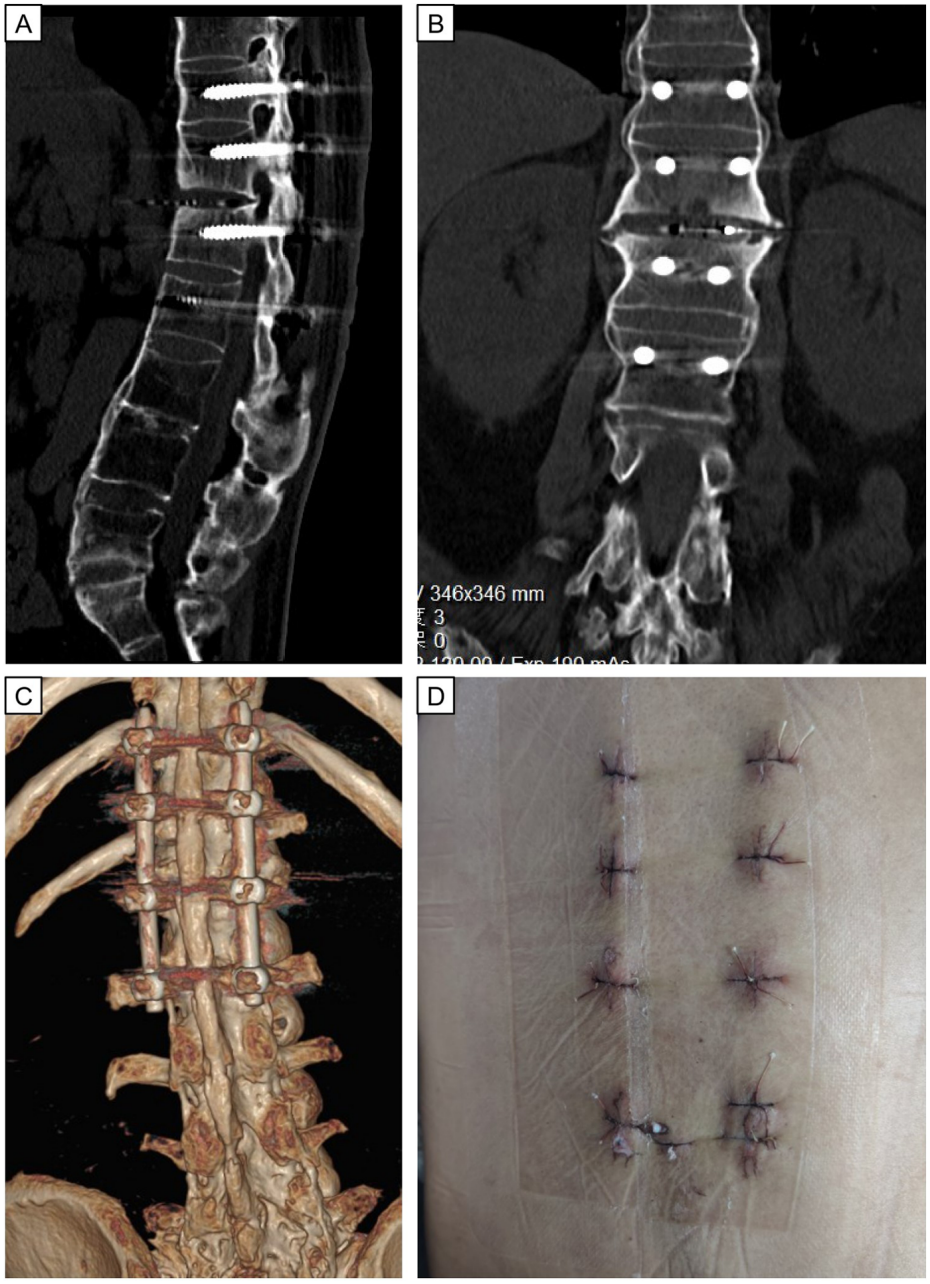
Postoperative immediate CT images and wound scars after minimally invasive surgery of LIF. Postoperative CT images **(A–C)** show good positioning of the internal fixation and normal sagittal sequence of the lumbar spine. Eight minimally invasive incisions **(D)** for internal fixation and fusion surgery.

Through LIF and posterior fixation of PPSs, spinal stability was improved and postoperative lower back pain was significantly alleviated. Compared with traditional open surgery, this surgical method had less blood loss and smaller incision wounds, which resulted in a faster recovery (Figure 4). We did not attempt to correct kyphosis through osteotomy, but *in situ* fusion can give the AL a biomechanically stable environment to heal.

## Discussion

An AL refers specifically to the destructive inflammatory lesion at the intervertebral disk-vertebral interface caused by AS. This scenario can affect the anterior, middle, and posterior columns, and it often occurs in the thoracolumbar region. In general, an AL is considered to be a rare complication in the late stage of AS. Due to the low incidence and lack of awareness of this lesion for most spinal surgeons, an AL is often misdiagnosed as infective spondylodiscitis, especially in geographic areas with a high incidence of tuberculosis. In this case, MRI data from a different hospital that was presented by the patient at the initial visit to our hospital influenced our initial diagnosis. Subsequent radiographic and CT findings revealed that the patient had AS with an AL.

Conservative treatment, including drugs (e.g., NSAIDs and anti-tumor necrosis factor-α agents), braces, rest, and physiotherapy, is feasible if symptoms are mild or absent (1–3). Etanercept, a dimeric fusion protein that inhibits the activity of TNF-α, has shown efficacy in the treatment of ankylosing spondylitis (4). Research indicates that certain single nucleotide polymorphisms (SNPs) in the TNF-α promoter can predict etanercept treatment response, aiding in personalized treatment based on genetic differences (4, 5). If the diagnosis is correct and the pain is unbearable, then decompression fusion and internal fixation surgery can be undertaken (as in our case) (6). The main purpose of the surgical procedure is to rebuild the stability of the spine and promote fusion of the spinal lesion, thereby alleviating lower back pain (6, 7). Various open surgical procedures through anterior, posterior, or combined approaches have been proposed (8–14). Fixation using PPSs can be considered only if axial back pain is present and only *in situ* fixation is required (15, 16). C. Zhang et al.'s recent study investigates the clinical and radiological outcomes of early MIS vs. open spinal fusion (OSF) for the treatment of AL, suggesting that MIS offers comparable efficacy to OSF with the advantages of reduced blood loss and shorter operation times (17). However, reports of minimally invasive cases that necessitate decompression, fusion, and percutaneous fixation are scarce. The most fundamental reason may arise from the characteristics of the disease: the rapidly growing and hard bone blurs the surgical anatomic structure, thereby increasing the risk of accidental injury to nerves and blood vessels. Moreover, outdated devices used for minimally invasive surgery have hampered endoscopic surgery. Recently, Zhou et al. showed that oblique lateral interbody fusion combined with posterior pedicle-screw implantation through the Wiltse paraspinal approach could be undertaken in a minimally invasive fusion for patients diagnosed with an AL (18). Compared to MIS tubular technology, the utilization of UBE provides a more direct and minimally invasive approach to spinal interventions, which is especially advantageous given the distinct anatomical considerations of our patient cohort. This technique facilitates enhanced visualization and manipulation within the spinal canal, a critical factor for the successful execution of the procedures undertaken. In brief, this is the first case report of a fully endoscopic LIF using UBE to treat an AL.

In this case, there are four main considerations when using UBE for LIF. First, due to the large number of osteophytes in pedicle attachments and poor imaging rendered by C-arm fluoroscopy, precise positioning and puncture of the pedicle are necessary before intraoperative insertion of pedicle wires. Second, a Kirschner wire must be employed to locate the lesion before endoscopy-assisted decompression and fusion. Third, considering that patients with AS have a faster rate of bone formation than other healthy counterparts, the amount of bone grafting can be reduced appropriately. Fourth, deep overextension should be avoided when inserting the interbody fusion cage because it can lead to damage of blood vessels/organs due to ossification and rupture of the anterior longitudinal ligament.

Precise preoperative planning can be aided by 3D printing technology. Due to challenges from posterior osteophytes, we pre-planned the positions, angles, and lengths of eight pedicle screws using a 3D-printed model. The model also helped us determine the curvature and length of titanium rods, overcoming stiffness and deformity in the patient's back. This preoperative planning ensured safe instrumentation and decompression, while also reducing surgery time.

This study is subject to certain limitations. Notably, the sample size was relatively small due to the rarity of AL as a complication of ankylosing spondylitis. Long-term follow-up is essential for evaluating the sustained efficacy of the treatment protocol. In future clinical studies, we intend to expand our case series and incorporate medium- to long-term follow-up assessments to enhance the robustness of our conclusions. Simultaneously, due to the limited number of surgical cases, we did not observe any potential surgical complications. As we all know, every surgery carries risks, and understanding these is crucial for the technology's development. From past experience, complications may include vascular or spinal cord injury, internal fixation loosening, fusion failure, and adjacent segment degeneration.

## Conclusions

Accurate diagnosis of an AL and AS is the foundation of treatment. Once the diagnosis has been established, if the patient has unbearable pain or symptoms indicative of neurological damage, then posterior fusion can be considered. Compared with open surgical procedures, combining UBE and 3D-printing technologies for fully endoscopic LIF can offer the advantages of minimal trauma, minimal bleeding, the same effect as open surgery, and faster postoperative recovery. Accurate preoperative planning based on a 3D-printing model is strongly recommended for patients suffering from an AL with ambiguous anatomic landmarks.

## Data Availability

The original contributions presented in the study are included in the article/Supplementary Material, further inquiries can be directed to the corresponding author.
